# Validation of Anthropometric Indices of Adiposity against Whole-Body Magnetic Resonance Imaging – A Study within the German European Prospective Investigation into Cancer and Nutrition (EPIC) Cohorts

**DOI:** 10.1371/journal.pone.0091586

**Published:** 2014-03-13

**Authors:** Jasmine Neamat-Allah, Diana Wald, Anika Hüsing, Birgit Teucher, Andrea Wendt, Stefan Delorme, Julien Dinkel, Matthaeus Vigl, Manuela M. Bergmann, Silke Feller, Johannes Hierholzer, Heiner Boeing, Rudolf Kaaks

**Affiliations:** 1 Division of Cancer Epidemiology, German Cancer Research Center, Heidelberg, Germany; 2 Division of Medical and Biological Informatics, German Cancer Research Center, Heidelberg, Germany; 3 Department of Translational Pulmonology, Translational Lung Research Center, Member of the German Center for Lung Research, University of Heidelberg, Heidelberg, Germany; 4 Division of Radiology, German Cancer Research Center, Heidelberg, Germany; 5 Department of Diagnostic and Interventional Radiology, University Hospital Heidelberg, Heidelberg, Germany; 6 Department of Epidemiology, German Institute of Human Nutrition Potsdam-Rehbrücke, Nuthetal, Germany; 7 Department of Diagnostic and Interventional Radiology, Clinic Ernst-von-Bergmann, Potsdam, Germany; University of Warwick – Medical School, United Kingdom

## Abstract

**Background:**

In epidemiological studies, measures of body fat generally are obtained through anthropometric indices such as the body mass index (BMI), waist (WC), and hip circumferences (HC). Such indices, however, can only provide estimates of a person’s true body fat content, overall or by adipose compartment, and may have limited accuracy, especially for the visceral adipose compartment (VAT).

**Objective:**

To determine the extent to which different body adipose tissue compartments are adequately predicted by anthropometry, and to identify anthropometric measures alone, or in combination to predict overall adiposity and specific adipose tissue compartments, independently of age and body size (height).

**Methods:**

In a sub-study of 1,192 participants of the German EPIC (European Prospective Investigation into Cancer and Nutrition) cohorts, whole-body MRI was performed to determine adipose and muscle tissue compartments. Additional anthropometric measurements of BMI, WC and HC were taken.

**Results:**

After adjusting for age and height, BMI, WC and HC were better predictors of total body volume (TBV), total adipose tissue (TAT) and subcutaneous adipose tissue (SAT) than for VAT, coronary adipose tissue (CAT) and skeletal muscle tissue (SMT). In both sexes, BMI was the best predictor for TBV (men: r = 0.72 [0.68–0.76], women: r = 0.80 [0.77–0.83]) and SMT (men: r = 0.52 [0.45–0.57], women: r = 0.48 [0.41–0.54]). WC was the best predictor variable for TAT (r = 0.48 [0.41–0.54]), VAT (r = 0.44 [0.37–0.50]) and CAT (r = 0.34 [0.26–0.41]) (men), and for VAT (r = 0.42 [0.35–0.49]) and CAT (r = 0.29 [0.22–0.37]) (women). BMI was the best predictor for TAT (r = 0.49 [0.43–0.55]) (women). HC was the best predictor for SAT (men (r = 0.39 [0.32–0.45]) and women (r = 0.52 [0.46–0.58])).

**Conclusions:**

Especially the volumes of internal body fat compartments are poorly predicted by anthropometry. A possible implication may be that associations of chronic disease risks with the sizes of internal body fat as measured by BMI, WC and HC may be strongly underestimated.

## Introduction

Excess body fat is known to be one of the main risk factors for diabetes, as well as cardiovascular diseases [1], [2] and several frequent types of cancer [3], [4]. In epidemiological studies addressing associations between excess body fat and disease risk, excess adipose tissue is most often estimated using the body mass index (BMI) [5], [6]. In addition, circumference measurements, waist circumference (WC) in particular, are used to improve estimates of visceral fat [6], [7] – a strong and recognized determinant of the metabolic syndrome and the risk of cardiovascular diseases, cancer, and mortality [8]–[10]. However, while the use of body circumference measures may help to improve estimates of visceral fat and related disease risks, these measurements may not allow an accurate differentiation between visceral and abdominal subcutaneous fat. A number of observations indicate that there can be a substantial part of “hidden” adiposity not properly accounted for by indices such as the BMI, suggesting a non-negligible prevalence of the metabolic syndrome, cardiovascular disease and cancer risk even for subjects whose BMI is still within the ‘normal’ range of 18–25 kg/m^2^ [11]. In addition, it is unclear whether standard anthropometric measurements allow an accurate estimation of the total amount of lean tissue.

Imprecision in measurements of body fat and lean tissue compartments is likely to result in misclassification of individuals by total body fat, and by size of visceral or subcutaneous fat, both overall and relative to the amount of lean body mass. As to a large extent this misclassification may be random, it will likely lead to an underestimation of relative risks of disease in relation to body composition [12]. To assess the possible magnitude of such underestimation, we conducted a large-scale validation study (N = 1,192) using MRI as a reference method. The aim of the validation sub-study, which was embedded within the two German sub-cohorts of the European Prospective into Cancer and Nutrition (EPIC)-study [13], was to allow a re-calibration of anthropometric measurements in order to be able to correct relative risk estimates of chronic diseases based on anthropometry in the German EPIC cohorts.

## Subjects and Methods

### Ethics Statement

The validation sub-study was approved by the local ethics committees in Potsdam and Heidelberg and all participants gave their written informed consent.

### EPIC-cohorts

The European Prospective Investigation into Cancer and Nutrition is a multi-center prospective cohort study designed to investigate the relation between diet, nutritional and metabolic characteristics, various lifestyle factors and the risk of chronic diseases in ten European countries with more than 520,000 participants [14]. The German EPIC cohorts consist of 25,540 participants from Heidelberg and 27,468 participants from Potsdam recruited between 1994 and 1998, and mostly aged between 35 and 65 years at both centers [13]. Over 98% of the EPIC study participants, who all entered the EPIC-Potsdam and EPIC-Heidelberg study in the 1990’s, were of European descent. At baseline, weight, height, and waist and hip circumferences (HC) were measured according to standardized protocols in both study centers by trained staff. In addition, participants were asked to fill in questionnaires about their educational level, their physical activity and their smoking habits.

### Selection of Cohort Participants for the Validation Sub-study

During 2010–2012, in which 21,864 cohort members in Heidelberg and 23,881 cohort members in Potsdam were still actively participating in the follow-up, a sub-sample of 1,234 participants from the two German EPIC cohorts was recruited to perform whole body magnetic resonance imaging (MRI) scans for the quantification of adipose and lean tissue compartments. The sub-study participants were recruited randomly according to a rectangular sampling scheme, designed to include approximately 300 men and 300 women in each of the two centers with equal representation of each of the three 10-year categories for age at baseline (35–44 years, 45–54 years and 55–64 years). The participation rates for the sub-study were 47% for Heidelberg and 55% for Potsdam. In addition to the whole body MRI scans, anthropometric measures were performed by trained staff according to standardized protocols.

People with metal implants such as cardiac pacemakers, defibrillators, stents, subcutaneous chips, tattoos, people with dementia, hemophilia, claustrophobia, a BMI>42, people under dialysis or with serious diseases diagnosed in the last 12 months and pregnant women were excluded from this study because of contraindications to MRI. Due to artifacts and image quality problems, 42 (3%) MRI datasets could not be analyzed in the two centers resulting in a final dataset of 1,192 participants (598 Heidelberg/594 Potsdam).

### Anthropometry

Anthropometric measurements, namely height, weight, waist and hip circumferences were measured in each subject of the sub-study by trained staff. All measurements were performed on study participants wearing nothing or at most light underwear and without shoes. BMI [kg/m^2^] was then calculated from these measurements. Weight was measured on a calibrated digital balance to the nearest 0.1 kg. Height was measured to the nearest 0.1 cm. WC was measured at midpoint between the distal border of the lowest rib and the superior border of the iliac crest and was measured to the nearest 0.1 cm. HC was measured at the widest point of the buttocks and was measured to the nearest 0.1 cm. In order to minimize measurement errors, waist and hip circumferences were measured 3 times and a mean value was calculated.

### Whole-body Magnetic Resonance Imaging

MRI examinations were performed with Siemens 1.5T Avanto MRI scanners (Erlangen, Germany) in Potsdam and Heidelberg using a 2-point Dixon technique with a 3D gradient echo sequence. The protocol used for the segmentation and quantification of body compartments is described in detail in Wald et al. [15].

During data acquisition, coils placed around the entire body were used to maximize the signal-to-noise ratio and to allow for high-resolution 3D parallel acquisition.

Due to the hardware limitation of the field-of-view of the MRI device, and due to elderly subjects not being able to hold their arms in a stable position above the head for the duration of the MRI protocol (10–12 minutes), the arms were positioned alongside the body instead. All participants were imaged in supine position. Wedges were used to prevent the arms aligning with the body. Total examination time was less than 15 minutes including positioning and placing the phase-array surface coils.

Due to the image field restrictions this meant that the arms were often partly or even completely outside the image area. For the purpose of standardization, we therefore decided to discard all image parts lateral to the armpits during data processing to ensure a consistent approach to body volume assessment for all participants.

### Segmentation of Adipose Tissue and Skeletal Muscle Tissue

The Dixon MRI protocol generates a fat and water image. Adipose tissue is shown brightly in the fat image, while other tissues appear dark. Thus, a simple threshold is sufficient to differentiate between adipose and non-adipose tissues, allowing the use of automated algorithms for image analyses and for the calculation of volumes for different body fat compartments, i.e. visceral (VAT), coronary (CAT), and subcutaneous adipose tissue (SAT). VAT is anatomically defined as the area within the muscular borders of the abdomen and vertically between the pelvic floor and the diaphragm. Coronary fat is found around the pericardium and the major blood vessels. SAT is located directly beneath the skin. Since all adipose tissue compartments have the same image intensity in the fat image, the separation was based on the specific location of each adipose tissue compartment in the body. For this purpose, statistical shape models were used to perform the classification of visceral adipose tissue in the abdomen and coronary adipose tissue in the thorax. All remaining adipose tissue, outside VAT and CAT, was scored as subcutaneous adipose tissue, while fat-containing bone marrow and intramuscular fat were removed in a post-processing step using standard image processing techniques of morphological operators and connected components analysis. The amount of total adipose tissue (TAT) (excluding bone marrow) was calculated by adding segmentations obtained for visceral, coronary and subcutaneous adipose tissue. Skeletal muscle tissue (SMT) forms the largest fraction of lean tissue in the human body, while lean tissue represents the body mass excluding adipose tissue, bones and air within hollow organs. Because lean tissue is shown brightly in the water image, a threshold was used to segment all lean tissue parts in the whole-body water image. In a subsequent step, the non-skeletal muscle tissue such as organ tissue, organ muscle or blood was removed using an abdomen and thorax mask, which was obtained as an intermediate result from the shape model segmentation of visceral and coronary adipose tissue [16].

The segmentation method was applied to 1,192 datasets (598 Heidelberg/594 Potsdam) and results were visually inspected. For quantitative analysis, segmentations of adipose and skeletal muscle tissue obtained with the automatic method were compared with ground truth segmentations created manually by an experienced operator. The results showed a significant agreement between the automatic and manual method. For further details of the segmentation method and evaluation, we refer to Wald et al. [15], [16].

**Figure 1:**
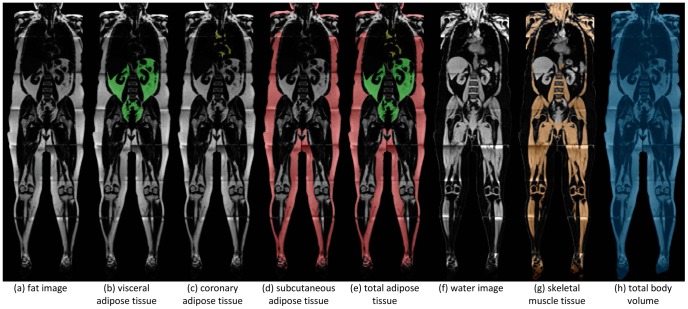
a–f. Illustration of different MRI body compartments in the sub-study of the German EPIC cohorts.

Figures **1a–f** show the different body compartments as assessed by MRI. The volumes of SAT, VAT and CAT add up to a total volume of adipose tissue (aggregate variable). Besides the total adipose tissue and its sub-compartments, the amount of SMT was also determined. TAT, SMT, and a rest volume consisting of bones, organs, liquids, and air within the lungs add up to the total body volume (TBV), which does not, however, include the head (not scanned by our MRI method) and arms (as explained above).

### Statistical Analysis

Descriptive statistics stratified by sex and age groups include mean values and ranges for all 1,192 participants. For anthropometric and MRI-based variables, means and ranges were very similar for Heidelberg and Potsdam. Therefore, it was decided to not do any further statistical analyses separately by center; by contrast, all analyses were performed separately for men and women. Correlation matrices were used to assess the relationship across anthropometric variables, between anthropometric and MRI variables and across MRI variables. Multiple linear regression analyses with BMI, waist, and hip circumferences as continuous predictor variables were used to estimate the proportion of between-subject variance of different MRI body compartments that could be explained by anthropometry. For all regression models, possible deviations from linearity in relationships between predictor and outcome variables were systematically investigated by visual assessment. Multiple linear regression analyses were further applied to identify the best anthropometric predictors of different body compartments as measured by MRI. To examine which of the three anthropometric variables – BMI, WC or HC – was the strongest predictor for each of the MRI-based measurements, partial correlations (type II sum of squares), where each predictor is adjusted for all other predictor variables in the model, were assessed. Predictor and outcome variables were adjusted for age and height with the residual method [17]. Using this method, regression analyses were used to compute residuals of MRI variables and anthropometric variables by removing the variation caused by height and age from each variable. For this purpose, all variables (MRI and anthropometric variables) were regressed on age and height in our analyses. As BMI, which by definition is already adjusted for height, also showed low correlations with height (r = 0.2) in the sex-stratified analyses, we also adjusted BMI for height with the residual method. The variance inflation factor (VIF) was used to detect multicollinearity between variables. However, as the VIF was below 6 for all variables, we assumed our variables to have a low degree of multicollinearity. Bootstrap validation with 100 bootstrap samples was used to examine the degree of overfitting of the models. Additionally, we investigated whether the inclusion of weight difference since age 18 (Heidelberg) and age 20 (Potsdam) as predictor variables, respectively, could further improve the prediction of the different body compartments. This variable had missing values for 97 participants. As this question was asked at different time points in Heidelberg and Potsdam and in order to adjust for possible recall biases, this variable was adjusted for age at interview (baseline age for Potsdam and age at follow-up 4 for Heidelberg) and was also adjusted for height with the residual method. All other variables (predictors and outcomes) had complete measurements for all 1,192 participants. The statistical analyses were conducted using the Statistical Analysis System (SAS) software package, Version 9.2 (SAS Institute Inc., Cary, NC) and R, version 4.1-0 (the R foundation for Statistical Computing, Vienna, Austria), package rms.

## Results

The overall distribution of all 1,192 sub-study participants by sex and age at baseline is shown in Table 1.

**Table 1:** Anthropometric variables and body compartments as assessed by MRI by sex and age groups^1^, all values are presented as mean (min, max).

	Men			Women		
Age group	51–61	58–71	68–81	47–61	58–71	68–81
No. of participants	191	194	213	207	196	191
**Anthropometry**						
Weight, *kg*	85.9 (53.4,128.5)	85.5 (55.6,125.9)	80.8 (56.9,113.5)	71.0 (44.4,116.2)	70.3 (43.9,116.7)	68.6 (42.1,107.0)
Height, *cm*	177.6 (160.0,197.5)	175.5 (156.9,198.6)	173.2 (155.2,191.1)	164.8 (152.0,183.1)	162.5 (142.5,180.2)	161.3 (147.1,179.0)
BMI, *kg/m^2^*	27.2 (18.6,40.8)	27.7 (20.2,38.3)	26.9 (19.1,38.9)	26.2 (17.1,40.8)	26.6 (18.0,42.1)	26.3 (17.1,40.5)
Waist, *cm*	99.2 (73.3,130.3)	102.1 (82.9,139.7)	101.1 (74.3,130.6)	88.9 (63.6,126.0)	91.3 (66.2,128.3)	90.9 (63.6,119.0)
Hip, *cm*	101.8 (85.5,123.0)	102.2 (78.3,122.0)	101.1 (86.9,127.1)	103.3 (80.0,137.7)	104.2 (84.8,141.2)	103.3 (83.6,129.6)
**MRI body compartments**						
TBV, *l*	73.3 (48.2,110.0)	73.8 (48.4,109.4)	70.1 (50.4,98.5)	63.4 (38.2,104.1)	63.4 (37.5,110.6)	61.8 (37.1,95.3)
TAT, *l*	20.7 (4.9,45.5)	22.1 (6.6,41.0)	20.9 (5.2,42.5)	24.3 (6.4,50.9)	25.1 (9.4,62.7)	24.0 (8.3,44.1)
VAT, *l*	4.8 (0.48,11.5)	5.4 (1.39,10.5)	5.5 (0.24,10.5)	2.7 (0.30,7.3)	3.0 (0.47,7.5)	3.2 (0.63,7.9)
SAT, *l*	15.5 (3.9,36.6)	16.1 (5.1,34.3)	14.9 (4.9,34.9)	21.4 (5.9,43.6)	21.7 (8.2,56.4)	20.5 (7.2,36.9)
CAT, *l*	0.42 (0.07,0.96)	0.50 (0.10,1.1)	0.51 (0.10,1.5)	0.24 (0.01,0.60)	0.31 (0.04,1.2)	0.32 (0.04,1.3)
SMT, *l*	25.4 (16.6,35.4)	24.3 (17.9,33.1)	22.6 (16.4,29.5)	16.9 (11.4,23.3)	16.2 (11.2,22.4)	15.8 (10.7,20.7)

TBV = total body volume, TAT = total adipose tissue, VAT = visceral adipose tissue, SAT = subcutaneous adipose tissue, CAT = coronary adipose tissue, SMT = skeletal muscle tissue.

1 Sub-study participants were sampled by baseline age groups (35–44 y, 45–54 y, 55–64 y). Due to the 4-year baseline period (1994–1998), age groups at time of sub-study (2010–2012) may overlap.

### Anthropometric Measurements and MRI Compartments by Sex and Age Group

With regard to anthropometry, men had a higher mean weight, body mass index and waist circumference than women, whereas women on average had a higher hip circumference. When considering the age groups, the middle age category (58–71 years at the time of participation in the sub-study) showed highest values for BMI, waist and hip circumferences among both men and women. By contrast, weight and height showed the highest values in the youngest age group (51–62 years in men and 47–62 years in women) and the lowest values in the highest age group (68–81 years) in both sexes.

For the MRI-based measurements, among both men and women, the largest volumes of TAT and SAT were seen in the middle age category [58–71 years]. By contrast, VAT and CAT were highest in the oldest age category (68–81 years) in both men and women, whereas SMT showed a progressive decrease from the youngest (51–61 years in men and 47–61 years in women) to the oldest age category (68–81 years) for both men and women (Table **1**).

### Correlations between Anthropometric Indices and MRI-based Measures of Adiposity

An examination of correlation matrices of the MRI-based plus anthropometric measurements, with all variables adjusted for age and height by the residual method, showed very similar patterns for men and women (Tables **2** and **3**). In both sexes, very high correlations (all above 0.82) were observed across the anthropometric indices of BMI, waist and hip circumferences. Likewise, within the variables of MRI-based measurements very high correlations were observed between the measures for TBV, TAT and SAT; all above 0.90 among men and above 0.96 among women. Between the anthropometric measurements and the MRI-based measurements, highest correlations were seen in both sexes for BMI, waist and hip circumferences with TBV, TAT and SAT (r>0.84 in men and r>0.85 in women). Lower correlations were observed between the internal body fat compartments (VAT, CAT), SMT and the anthropometric indices. VAT showed the highest correlations with waist circumference (r = 0.80 [95% CI: 0.77–0.83] in men and women), and somewhat weaker correlations with BMI (0.75 [0.72–0.79] in men; 0.77 [0.73–0.80] in women) and hip circumference (0.64 [0.59–0.68] in men; 0.70 [0.65–0.74] in women). Similarly, CAT also showed highest correlations with waist circumference (0.70 [0.65–0.74] in men; 0.65 [0.60–0.69] in women). For SMT, BMI was the anthropometric index showing strongest correlations among both men (0.63 [0.58–0.67]) and women (0.69 [0.65–0.73]).

**Table 2:** Pearson correlation coefficients (95% CI) between anthropometric and MRI variables adjusted for age and height with the residual method in men (n = 598).

	BMI	WC	HC	TBV	TAT	SAT	VAT	CAT
**WC**	**0.91** (0.89–0.92)							
**HC**	**0.86** (0.83–0.88)	**0.82** (0.80–0.85)						
**TBV**	**0.96** (0.96–0.97)	**0.92** (0.90–0.93)	**0.87** (0.85–0.89)					
**TAT**	**0.89** (0.87–0.91)	**0.91** (0.89–0.92)	**0.84** (0.82–0.86)	**0.92** (0.91–0.93)				
**SAT**	**0.87** (0.85–0.89)	**0.87** (0.85–0.89)	**0.85** (0.83–0.87)	**0.90** (0.88–0.91)	**0.97** (0.97–0.98)			
**VAT**	**0.75** (0.72–0.79)	**0.80** (0.77–0.83)	**0.64** (0.59–0.68)	**0.78** (0.75–0.81)	**0.85** (0.82–0.87)	**0.70** (0.66–0.74)		
**CAT**	**0.65** (0.60–0.70)	**0.70** (0.65–0.74)	**0.55** (0.50–0.61)	**0.67** (0.62–0.71)	**0.75** (0.72–0.79)	**0.65** (0.60–0.69)	**0.80** (0.76–0.82)	
**SMT**	**0.63** (0.58–0.67)	**0.49** (0.42–0.54)	**0.46** (0.40–0.52)	**0.62** (0.57–0.67)	**0.33** (0.25–0.40)	**0.28** (0.20–0.35)	**0.38** (0.31–0.44)	**0.24** (0.16–0.31)

BMI = body mass index, WC = waist circumference, HC = hip circumference, TBV = total body volume, TAT = total adipose tissue, SAT = subcutaneous adipose tissue, VAT = visceral adipose tissue, CAT = coronary adipose tissue, SMT = skeletal muscle tissue.

**Table 3:** Pearson correlation coefficients (95% CI) between anthropometric and MRI variables adjusted for age and height with the residual method in women (n = 594).

	BMI	WC	HC	TBV	TAT	SAT	VAT	CAT
**WC**	**0.89** (0.88–0.91)							
**HC**	**0.93** (0.92–0.94)	**0.85** (0.83–0.87)						
**TBV**	**0.98** (0.98–0.98)	**0.89** (0.88–0.91)	**0.94** (0.93–0.95)					
**TAT**	**0.95** (0.94–0.96)	**0.88** (0.86–0.90)	**0.94** (0.93–0.95)	**0.97** (0.96–0.97)				
**SAT**	**0.94** (0.93–0.95)	**0.85** (0.83–0.87)	**0.94** (0.93–0.95)	**0.96** (0.95–0.96)	**0.99** (0.99–0.99)			
**VAT**	**0.77** (0.73–0.80)	**0.80** (0.77–0.83)	**0.70** (0.65–0.74)	**0.78** (0.75–0.81)	**0.80** (0.77–0.83)	**0.71** (0.67–0.75)		
**CAT**	**0.61** (0.56–0.66)	**0.65** (0.60–0.69)	**0.56** (0.50–0.61)	**0.62** (0.57–0.67)	**0.64** (0.59–0.68)	**0.57** (0.51–0.62)	**0.75** (0.72–0.79)	
**SMT**	**0.69** (0.65–0.73)	**0.63** (0.57–0.67)	**0.56** (0.50–0.61)	**0.68** (0.63–0.72)	**0.53** (0.47–0.58)	**0.50** (0.44–0.56)	**0.50** (0.44–0.56)	**0.39** (0.32–0.46)

BMI = body mass index, WC = waist circumference, HC = hip circumference, TBV = total body volume, TAT = total adipose tissue, SAT = subcutaneous adipose tissue, VAT = visceral adipose tissue, CAT = coronary adipose tissue, SMT = skeletal muscle tissue.

### Multiple Linear Regression Analyses

In multivariate regression models, after adjustment for age and body height, over 82% and over 91% of the variance in TBV, TAT and SAT in men and women, respectively, could be predicted by the variables of BMI, waist and hip circumferences. By contrast, only 65% (men) and 67% (women) of the variance in VAT and around 45% of the variance in CAT could be explained by these predictors in men and women. For SMT, 45% (men) and 53% (women) of the variance could be explained by the anthropometric variables. The predictive equations of body compartments after adjustment for age and height (Table **S1 in** **File S1**) can be found in Table **S2 in** **File S1**.

**Figure 2:**
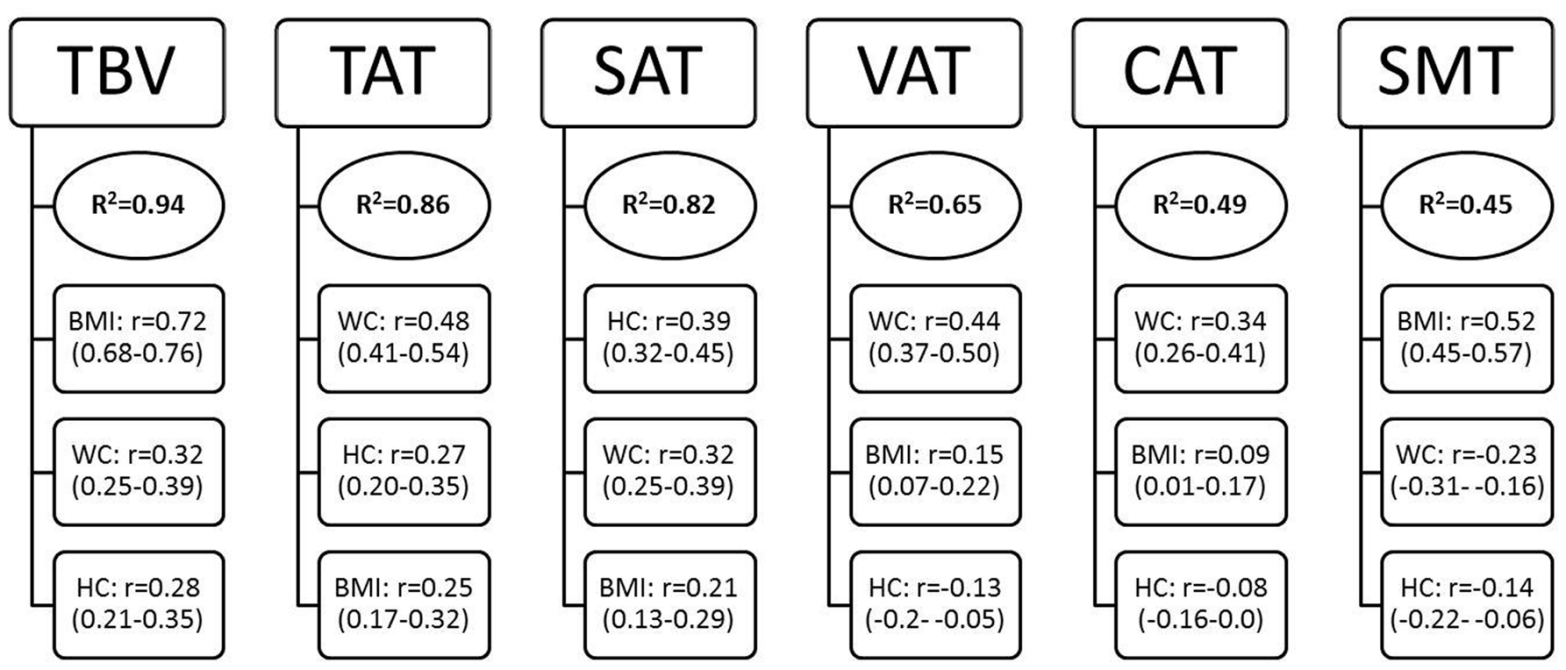
Prediction of body compartments by anthropometric indices in multiple linear regression analyses (Men, n = 598). Total model R^2^ for each body compartment and partial correlation coefficients (95% CI) for anthropometric indices. All variables were adjusted for age and height. TBV = Total body volume, TAT = total adipose tissue, SAT = subcutaneous adipose tissue, VAT = visceral adipose tissue, CAT = coronary adipose tissue, SMT = skeletal muscle tissue, BMI = body mass index, WC = waist circumference, HC = hip circumference. ^1^ Predictors included: BMI, WC, HC. All variables (predictors and outcome) adjusted by age and height with the residual method. ^2^Partial correlation coefficients (95% CI) are reported for predictor variables.

**Figure 3:**
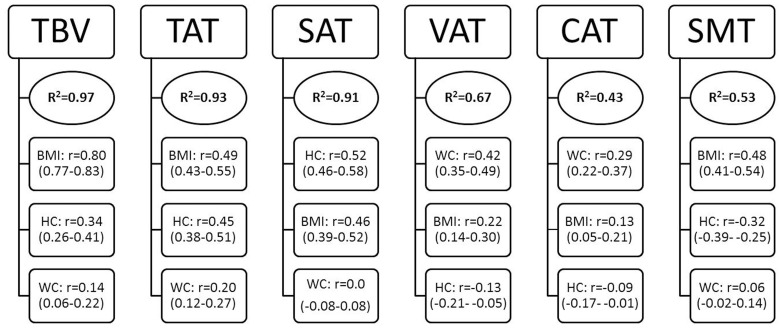
Prediction of body compartments by anthropometric indices in multiple linear regression analyses (Women, n = 594). Total model R^2^ for each body compartment and partial correlation coefficients (95% CI) for anthropometric indices. All variables were adjusted for age and height. TBV = Total body volume, TAT = total adipose tissue, SAT = subcutaneous adipose tissue, VAT = visceral adipose tissue, CAT = coronary adipose tissue, SMT = skeletal muscle tissue, BMI = body mass index, WC = waist circumference, HC = hip circumference. ^1^ Predictors included: BMI, WC, HC. All variables (predictors and outcome) adjusted by age and height with the residual method. ^2^Partial correlation coefficients (95% CI) are reported for predictor variables.

Analyses of partial correlations showed that, in both men and women, waist circumference was the best predictor variable for VAT (r = 0.44 [0.37–0.50] in men and r = 0.42 [0.35–0.49] in women) and CAT (r = 0.34 [0.26–0.41] in men and (r = 0.29 [0.22–0.37] in women). In both sexes, BMI was the strongest predictor for (height-and age-adjusted) TBV (r = 0.72 [0.68–0.76] in men and r = 0.80 [0.77–0.83] in women) and SMT (r = 0.52 [0.45–0.57] in men and r = 0.48 [0.41–0.54] in women), and hip circumference was the strongest predictor for SAT (r = 0.39 [0.32–0.45] in men, r = 0.52 [0.46–0.58] in women). The single best predictor for TAT was waist circumference in men (r = 0.48 [0.41–0.54]), and BMI in women (r = 0.49 [0.43–0.55]) (Figures **2** and **3**).

### Bootstrap Validation

Internal validation with 100 bootstrap samples produced corrected R^2^ about 1% lower than the calculated R^2^ for all body compartments (data not shown).

## Discussion

Using a large-scale validation study nested within the two German EPIC cohorts, we validated anthropometric measurements with the use of MRI measurements of different body compartments. Magnetic resonance imaging is a well-established tool for quantifying the sizes of adipose and lean tissue compartments [18], but is generally too costly for large-scale application and requires expensive equipment. Also, MRI analyses are often limited by operator-intensive and time-consuming processes for image analyses. Most previous studies using MRI for adiposity research had therefore included relatively few study participants and/or limited imaging analyses to small anatomical areas such as the abdominal area only [19], [20], or even to single image slices [21], [22]. By developing automated algorithms for the analysis of MRI images (for images based on the Dixon technique) [15], we were able to apply MRI for whole-body measurements of adipose and lean tissue compartments in a relatively large study of 1,192 study participants. The automated quantification algorithms showed robust results without any user interaction [15], and thus also had the advantage of avoiding subjective inter-observer biases.

We observed close to perfect correlations (>0.97) between the aggregate measures of TAT and SAT, and also VAT showed a relatively high correlation (>0.80) with TAT. Between the three distinctive adipose tissue compartments of VAT, CAT and SAT, however, the correlations were of a more modest magnitude (around 0.5–0.8), and skeletal muscle tissue volume showed correlations of only about 0.2–0.5 with each of these compartments. These latter, more moderate correlation values clearly document a certain degree of freedom for each basic adipose tissue compartment to have different and potentially specific relationships with risk of chronic diseases. It has long been recognized that the cardiovascular risk of adiposity may be more strongly related to a specific body fat distribution than to total body fat [23], and especially the clinical importance of VAT with regard to mechanisms leading to insulin resistance has been described in several studies [24], [25]. For coronary adipose tissue, a specific relationship with disease risk is also plausible, although so far there is less epidemiologic evidence documenting this hypothesis. Epicardial fat is located between the myocardium and visceral pericardium, whereas pericardial fat is situated outside the visceral pericardium and on the external surface of the parietal pericardium [26]. Compared to pericardial fat, epicardial fat has been reported as the metabolically more important compartment in previous studies as it correlates with coronary artery disease, insulin resistance and the metabolic syndrome [26]. In our study, due to limitations of our MRI method, we could report only on CAT, an aggregate measure of peri- and epicardial fat. Besides the internal fat deposits of VAT and CAT, the subcutaneous adipose tissue compartment may also contribute to disease risks (e.g. risks of endometrial cancers [27]) by specific effects on metabolism like peripheral estrogen synthesis [28].

For the optimal interpretation of the sizes of adipose tissue compartments with regard to possible impacts on metabolism and disease risk, it is essential to adjust the variables (predictors and outcome) for basic body size. In our present analysis, we used height as the measure of body “size”, in line with the fact that mathematically body weight scales to height with a power of about 2, thus forming the basis of BMI (weight/height^2^) as a “shape-for-size” index that is practically height (size) independent [29], [30]. An underlying assumption is that body fat and fat-free mass also scale proportionally to height squared [30], so that after height adjustment women or men for a given weight have approximately the same proportion of body weight as fat [29], [31]. In our analyses, BMI and body circumference measurements showed a notably better prediction capacity for the tissue compartments of VAT, CAT, SAT and SMT when all measurements were adjusted for height and age in comparison with the unadjusted model or the age-adjusted model, only. For instance, the proportion of variation explained, i.e. the model R^2^ of age- and height-adjusted skeletal muscle tissue (SMT) increased from 38.8% to 44.8% in men when comparing the models of unadjusted predictors with the models of age- and height-adjusted predictors (data not shown). In line with these results, one would also expect that, in epidemiologic analyses of disease risk, a statistical adjustment for height should improve the prediction of disease risk in relation to anthropometric indices of adiposity, including risk associations with waist and hip circumferences.

Adjusting for height and age, the three anthropometric indices of BMI, waist and hip circumferences showed to be very strong predictors of the more aggregate measures of whole-body adiposity (TAT) and total subcutaneous fat (SAT), but less strongly for the distinctive compartments of visceral and coronary adipose tissue and skeletal muscle volume. The strength of association between anthropometric indices and VAT in our study is similar to that reported in other studies [32]–[36], although some studies reported lower correlation coefficients [21], [37]–[39]. The single best anthropometric predictor for the specific measures of both VAT and CAT appeared to be waist circumference in both sexes, although BMI showed almost equally strong univariate correlations.

Overall, multivariate regression models including BMI plus waist and hip circumferences could explain up to 86 and 93 per cent of (height and age-adjusted) between-subject variation in TAT in men and women, respectively, and a similar magnitude of prediction was reached for SAT. By contrast, the prediction capacity for between-subject differences in VAT, CAT and SMT was substantially lower, with models explaining between 43 to 67 per cent of the variation. Internal validation via bootstrap showed that bias due to overfitting was negligible in our analyses.

In a number of epidemiologic studies, self-reported weight gain since early adulthood was found to predict chronic disease risks (e.g. of breast cancer, colorectal cancer) over and above measurements of BMI and body circumferences taken at a more advanced age [40], [41]. In our multivariate models, however, the inclusion of the variable weight gain since age of 18–20 years did not lead to any meaningful increase in the proportion of variation explained by the models to predict age-, sex- and height-standardized volumes of VAT, SAT, TAT or other compartments.

The present validation sub-study was conducted for the purpose of estimating the extent to which the associations of chronic disease risks (relative, attributable and absolute risk estimates) could be underestimated, when anthropometric measures, such as BMI, WC and/or HC are included in the models as a proxy for TAT, VAT and SAT. Although detailed analyses of this question with regard to the EPIC cohort data would go beyond the scope of the present report, basic calculations indicate that the relative and attributable risk estimates based on anthropometric data are likely to be attenuated, particularly for chronic diseases where the amount of visceral adipose tissue is etiologically relevant. Adjusting for height and age, our multivariate regression models could explain only about 65 per cent of between-subject variance in the volume of VAT, as indicated by the model R^2^ estimate. Estimates of relative risk per unit increase in VAT would be underestimated by a factor equal to this R^2^ measure, and this would also translate into an underestimation of attributable risks.

### Study Limitations

The present validation study, making use of whole-body MRI for the assessment of body composition, included a relatively large sample size as compared to previous studies with a good representation of men and women over the age range of 47–81 years in the two German EPIC cohorts. Our validation study results therefore apply to individuals of middle age and older. Had younger subjects been included, the associations between body compartments and anthropometric indices and our prediction models equations could have been somewhat different.

A possible limitation, however, may be that rates of participation in our sub-study was around 50% only, which may limit the interpretation of the sub-study in terms of representativeness for the full German EPIC cohort. However, variable distributions for BMI and waist circumference, as well as for important health-related covariates (physical activity, smoking status, socioeconomic status) were very similar between sub-study participants and other cohort members, suggesting that selection biases, if any, would be small.

As mentioned above, due to limitations of the MRI method chosen, arms were discarded from all whole-body images. Whole-body volume measurements did therefore not include the upper extremities and the head which leads to an underestimation of the true volume of all whole-volume compartments (TBV, TAT, SAT, SMT). However, we expect that, in terms of both absolute and relative volumes, the amounts of muscle tissue in the arms correlates well with those in the rest of the body and that the same applies to SAT. Therefore, we assume that the inclusion of the arms would not have considerably changed the results and associations found in our study.

### Conclusions

Compared to a gold standard method like MRI, in the age group of 47 to 81 years, the level of prediction of TAT and SAT by anthropometry is adequate. In men and women, WC provides slightly better predictions of VAT compared to BMI, but the overall prediction of VAT is limited. Considering that VAT plays a more pronounced role in chronic disease risk, and with only 65% of the variance in VAT predicted by anthropometric measures, relative risk estimates for chronic diseases may be substantially underestimated. Also the moderate correlation between VAT, CAT, and SAT, points at the existence of a certain degree of freedom for each basic adipose tissue compartment. In terms of understanding chronic disease risk in relation to specific body fat compartments and muscle mass, this needs to be addressed in further studies.

## Supporting Information

File S1 (DOCX)
