# Supplementary material for: Validation of Anthropometric Indices of Adiposity against Whole-Body Magnetic Resonance Imaging – A Study within the German European Prospective Investigation into Cancer and Nutrition (EPIC) Cohorts
Source: PLoS One. 2014 Mar 13;9(3):e91586. doi: 10.1371/journal.pone.0091586 (PMC3953447; doi:10.1371/journal.pone.0091586)
Supplement: File S1 — (DOCX) [file pone.0091586.s001.docx]

**Table S1. Estimates for adjustment for age and height with the residual method in men (n=598) and women (n=594).**

|  |  | **Height** | | **Age** | |
| --- | --- | --- | --- | --- | --- |
|  | Intercept | β | 95% CI | β | 95% CI |
| **Men** |  |  |  |  |  |
| TBV | -19.3 | 0.554 | (0.430 - 0.679) | -0.085 | (-0.196 - 0.027) |
| TAT | -0.95 | 0.119 | (0.037 - 0.201) | 0.020 | (-0.054 - 0.094) |
| SAT | 2.9 | 0.084 | (0.023 - 0.145) | -0.032 | (-0.087 - 0.023) |
| VAT | -4.0 | 0.035 | (0.010 - 0.061) | 0.047 | (0.024 - 0.070) |
| CAT | 0.71 | -0.000 | (-0.003 - 0.002) | 0.005 | (0.003 - 0.007) |
| SMT | 2.1 | 0.174 | (0.144 - 0.203) | -0.130 | (-0.156 - -0.103) |
| BMI | 47.4 | -0.068 | (-0.111 - -0.024) | -0.049 | (-0.089 - -0.010) |
| Waist | 64.7 | 0.169 | (0.041 - 0.298) | 0.098 | (-0.018 - 0.213) |
| Hip | 53.0 | 0.275 | (0.196 - 0.354) | 0.008 | (-0.063 - 0.079) |
| **Women** |  |  |  |  |  |
| TBV | -22.2 | 0.514 | (0.349 - 0.679) | 0.021 | (-0.092 - 0.135) |
| TAT | 0.47 | 0.139 | (0.024 - 0.255) | 0.020 | (-0.060 - 0.100) |
| SAT | 2.5 | 0.122 | (0.023 - 0.220) | -0.017 | (-0.085 - 0.051) |
| VAT | -1.9 | 0.017 | (-0.004 - 0.040) | 0.032 | (0.018 - 0.047) |
| CAT | -0.05 | 0.000 | (-0.002 - 0.002) | 0.004 | (0.003 - 0.006) |
| SMT | -6.8 | 0.154 | (0.129 - 0.179) | -0.029 | (-0.046 - -0.012) |
| BMI | 41.8 | -0.093 | (-0.157 - -0.030) | -0.003 | (-0.047 - 0.040) |
| Waist | 48.1 | 0.199 | (0.038 - 0.361) | 0.152 | (0.040 - 0.264) |
| Hip | 65.6 | 0.215 | (0.079 - 0.351) | 0.046 | (-0.048 - 0.139) |

TBV=total body volume, TAT=total adipose tissue, SAT=subcutaneous adipose tissue, VAT=visceral adipose tissue, CAT=coronary adipose tissue, SMT=skeletal muscle tissue, BMI=body mass index

**Table S2. Predictive equations for body compartments based on multiple linear regression analyses in men (n=598) and women (n=594), all measurements adjusted for height and age.**

|  |  | **BMI** | | **Waist** | | **Hip** | |
| --- | --- | --- | --- | --- | --- | --- | --- |
|  | Intercept | β | 95% CI | β | 95% CI | β | 95% CI |
| **Men** | | | | | | | |
| TBV | -3.1 *10^-14^ | 1.904 | (1.756 - 2.052) | 0.192 | (0.146 - 0.238) | 0.219 | (0.159 - 0.279) |
| TAT | 3.8 * 10^-14^ | 0.489 | (0.336 - 0.643) | 0.319 | (0.271 - 0.366) | 0.219 | (0.157 - 0.282) |
| SAT | 4.2 *10^-14^ | 0.345 | (0.216 - 0.472) | 0.168 | (0.129 - 0.208) | 0.270 | (0.218 - 0.322) |
| VAT | 9.0 *10^-16^ | 0.134 | (0.061 - 0.207) | 0.138 | (0.116 - 0.161) | -0.046 | (-0.076 - -0.017) |
| CAT | 6.3 * 10^-16^ | 0.010 | (0.002 - 0.019) | 0.012 | (0.009 - 0.015) | -0.004 | (-0.007 - -0.000) |
| SMT | -2.5 * 10^-14^ | 0.814 | (0.705 - 0.923) | -0.100 | (-0.133 - -0.066) | -0.079 | (-0.123 - -0.036) |
| **Women** | | | | | | | |
| TBV | 4.8 * 10^-14^ | 1.993 | (1.874 - 2.112) | 0.056 | (0.024 - 0.089) | 0.211 | (0.163 - 0.258) |
| TAT | 2.3 * 10^-15^ | 0.905 | (0.776 - 1.034) | 0.087 | (0.052 - 0.122) | 0.318 | (0.266 - 0.369) |
| SAT | -5.1 * 10^-15^ | 0.756 | (0.638 - 0.874) | 0.002 | (-0.030 - 0.034) | 0.354 | (0.306 - 0.401) |
| VAT | 3.3 * 10^-15^ | 0.139 | (0.089 - 0.189) | 0.079 | (0.065 - 0.092) | -0.033 | (-0.053 - -0.013) |
| CAT | 6.5 * 10^-16^ | 0.010 | (0.004 - 0.016) | 0.006 | (0.005 - 0.008) | -0.003 | (-0.005 - -0.000) |
| SMT | 2.2 * 10^-14^ | 0.478 | (0.406 - 0.550) | 0.016 | (-0.004 - 0.035) | -0.119 | (-0.147 - -0.090) |

TBV=total body volume, TAT=total adipose tissue, SAT=subcutaneous adipose tissue, VAT=visceral adipose tissue, CAT=coronary adipose tissue, SMT=skeletal muscle tissue, BMI=body mass index
